# MicroRNAs: Emerging Novel Clinical Biomarkers for Hepatocellular Carcinomas

**DOI:** 10.3390/jcm4081631

**Published:** 2015-08-18

**Authors:** Sumadi Lukman Anwar, Ulrich Lehmann

**Affiliations:** 1 Department of Surgery, Faculty of Medicine, Universitas Gadjah Mada, Yogyakarta 55281, Indonesia; 2 Institute of Pathology, Medizinische Hochschule Hannover, Hannover D30625, Germany

**Keywords:** microRNA, hepatocellular carcinoma, biomarker, diagnosis, prognosis, therapeutic monitoring

## Abstract

The discovery of small non-coding RNAs known as microRNAs has refined our view of the complexity of gene expression regulation. In hepatocellular carcinoma (HCC), the fifth most frequent cancer and the third leading cause of cancer death worldwide, dysregulation of microRNAs has been implicated in all aspects of hepatocarcinogenesis. In addition, alterations of microRNA expression have also been reported in non-cancerous liver diseases including chronic hepatitis and liver cirrhosis. MicroRNAs have been proposed as clinically useful diagnostic biomarkers to differentiate HCC from different liver pathologies and healthy controls. Unique patterns of microRNA expression have also been implicated as biomarkers for prognosis as well as to predict and monitor therapeutic responses in HCC. Since dysregulation has been detected in various specimens including primary liver cancer tissues, serum, plasma, and urine, microRNAs represent novel non-invasive markers for HCC screening and predicting therapeutic responses. However, despite a significant number of studies, a consensus on which microRNA panels, sample types, and methodologies for microRNA expression analysis have to be used has not yet been established. This review focuses on potential values, benefits, and limitations of microRNAs as new clinical markers for diagnosis, prognosis, prediction, and therapeutic monitoring in HCC.

## 1. Introduction

Human hepatocellular carcinoma (HCC) is the most common type of primary liver cancer, which ranks as the fifth most frequent cancer and the third leading cause of cancer mortality worldwide [1]. HCC is frequently diagnosed at a late stage in individuals with severe liver dysfunction, resulting in a high mortality rate and short overall survival [2]. These facts suggest that understanding the cellular and molecular mechanisms leading to full-blown malignant liver tumors is crucial in order to improve clinical outcomes as well as to develop early diagnostic markers and new therapeutic options for patients with HCC.

Liver cancer is a heterogeneous and complex disease that develops through step-wise accumulation of genetic and epigenetic alterations [3]. Genetic alterations such as mutations, translocation, gene deletions and amplifications have been established as major drivers in carcinogenesis [4]. Epigenetics refers to inherited modifications affecting gene expression and cellular phenotypes without involving any DNA sequence changes [5]. Epigenetics has also been reported as a key player during cancer initiation and progression and represent diverse processes affecting a broad range of cellular functions. Epigenetic mechanisms include several distinct and self-reinforcing processes such as DNA modifications, chromatin remodeling and non-coding RNAs [6]. Contained within the class of non-coding RNAs are microRNAs, which make up the best-studied class of non-coding RNAs. MicroRNAs are well-conserved, short, single-stranded RNA molecules (20–22 nucleotides) that negatively modulate gene transcription through binding to mRNA targets. Studies over the past decades highlight the magnitude of microRNA’s role as a key regulator of many important biological processes including cell proliferation, differentiation, apoptosis, and embryonic development [5].

Dysregulation of microRNA expression has been documented in almost every human cancer including HCC [7,8,9]. Unique patterns of microRNA expression have been established as a potential marker for sub-classification, diagnosis, prognosis, and therapeutic targets in HCC. However, each study reported a different panel, and only a few microRNAs are contained within more than a single panel. Possible reasons for this lack of concordance are different technologies for the analysis, variations of sample sources, and heterogeneity of the disease. Up till now, there has been no general consensus on which candidate microRNAs are potentially useful for diagnosis, prognosis, and prediction in HCC. To develop reliable biomarkers, robust laboratory assays, including precision, accuracy, reproducibility, and generalizability, are required [10]. In addition, large prospective and cross-sectional studies are required to validate the candidate biomarkers before entering the daily routine in the clinics. In this review, we focus on microRNAs that are established as biomarkers for diagnosis, prognosis, and prediction of therapeutic response. 

## 2. MicroRNA (miRNA)

**Figure 1:**
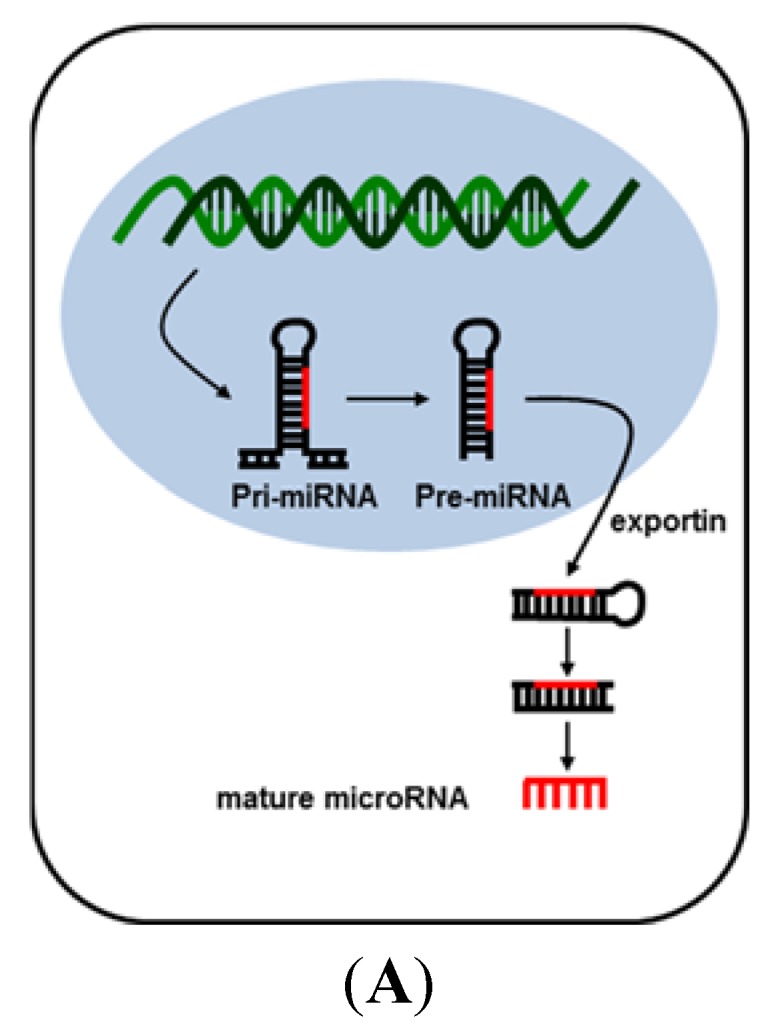

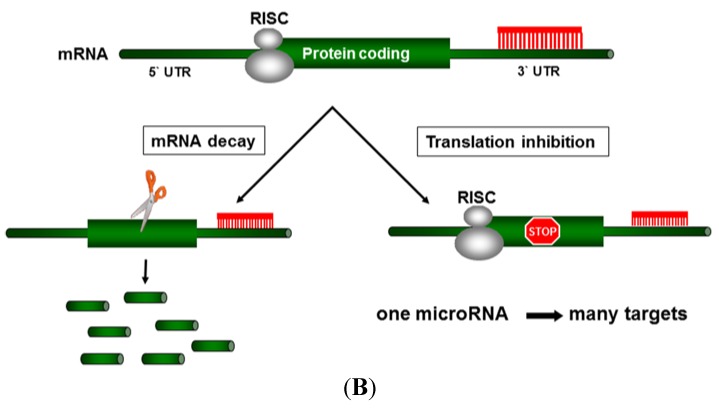
Biogenesis of microRNA (**A**) and transcriptional inhibition by microRNA (**B**). MicroRNA is transcribed from microRNA genes by RNA polymerase II into primordial-microRNAs. Segments of pri-miRNA contain a stem-loop structure that can be recognized by DiGeorge Syndrome Critical Region gene 8 (DGCR8) proteins for subsequent processing by RNase type III Drosha to produce pre-microRNAs. The hairpin-contained pre-microRNAs are then exported from the nucleus to the cytoplasm by a protein complex containing exportin-5 and RNA-GTP. In the cytoplasm, pre-microRNAs are further sliced by RNase type III Dicer, eliciting double-strand, ~22 nucleotide-long, mature microRNAs. After the duplex mature microRNA unwinds, degradation of the other strand follows. The single stranded mature microRNA within the RISC complex can subsequently act as a binding site to the messenger RNA (mRNA) targets. Perfect or nearly perfect complementarity to the 3′ UTR of mRNA results in cleavage of the mRNA targets. Partial complementarity of miRNA results in translational inhibition.

MicroRNAs are small non-coding RNAs that function as master regulators of gene expression [5,11]. They are primarily transcribed from microRNA genes by RNA polymerase II into several hundred- to thousand-bp-long primordial-microRNAs that are generally capped with a uniquely-modified base and polyadenylated at the tail [12]. Segments of pri-miRNA contain a stem-loop structure that can be recognized by DiGeorge Syndrome Critical Region gene 8 (DGCR8) proteins for subsequent processing by RNase type III Drosha to produce 65–100 bp long pre-microRNAs. The hairpin contained pre-microRNAs are then exported from the nucleus to the cytoplasm by a protein complex containing exportin-5 and RNA-GTP. In the cytoplasm, pre-microRNAs are further sliced by RNase type III Dicer, eliciting double-strand ~22 nucleotide-long mature microRNAs. These mature microRNAs are then incorporated into RNA-induced silencer complex (RISC). After the duplex mature microRNAs unwinds, degradation of the other strand follows. The single stranded mature microRNA within the RISC complex can subsequently act as a binding site for the messenger RNA (mRNA) targets. Argonaute (Ago) protein family plays a central role in the RISC complex. The PAZ (Piwi/Argonaute/Zwille) domain in Ago proteins is essential for binding to the 3′-end, while the PIWI domain is used to recognize the 5′-end of the guide strand. Perfect or nearly perfect complementarity to the 3′ UTR of mRNA results in cleavage of the mRNA targets. Ago family proteins are generally responsible for cleavage while SKI complex and XRN1 for degradation of target mRNAs [13,14]. However, Ago2 can directly cleave and degrade the mRNAs. On the other hand, partial complementarity of a miRNA to the target mRNA will induce translational inhibition through removal of the cap and adenyl-group from the mRNA target by means of interaction with DCP1-DCP2 and CAF1-CCR4-NOT protein complexes. Removal of the cap and adenyl group affects the mRNA stability [15] microRNAs are implicated to regulate up to 30% of the total human genes thus revealing that microRNAs are the most abundant regulators of gene expression in human [16]. Biogenesis of microRNA is depicted schematically in Figure 1.

By modulating gene expression post-transcriptionally, microRNAs play an important role in various basic biological processes such as embryonic development, cell cycle checkpoint, cell proliferation, migration, differentiation, and apoptosis [11]. It is therefore not surprising that dysregulation of microRNA expression is involved in a number of diseases including developmental disorders, neurological diseases, cardiovascular disorders, and cancer. First identified in 1993, the involvement of microRNAs in cancer was initially described in 2002 by Calin *et al.* [17]. MicroRNAs negatively regulate either oncogenes or tumor suppressor genes. Therefore, their role in oncogenesis can be either oncogenic or tumor-suppressive depending on their target genes and the cellular context.

## 3. MicroRNAs in Liver Carcinogenesis

A number of studies have documented frequent and extensive microRNA dysregulation in liver adenoma, cirrhosis, and different stages of liver cancer [18] Genomic changes [19] including deletion, amplification, mutations and epigenetic alterations including DNA methylation [20,21] and histone modification [22] can affect dysregulation of microRNA expression. Application of genome-wide expression analysis such as microarray and next-generation sequencing reveals more differentially regulated microRNAs in HCC. In addition, unique patterns of microRNA expression are valuable as potential markers for diagnosis, prognosis, staging, and prediction of therapeutic responses in HCC [23,24]. With a significant number of studies addressing microRNA dysregulation in HCC, differential microRNA expression in primary liver cancer samples has been comprehensively reported and reviewed [8,25,26] MiR-17-92 cluster, miR-21, miR-221, miR-222, and miR-224 are consistently up-regulated in primary HCC samples [8,27]. On the other hand, members of the let-7 and miR-200 families, as well as miR-29, miR-122, miR-124, and miR-199a/b, are commonly downregulated in HCC [8,25] and MiR-24 and miR-27a were commonly downregulated in HCCs with cirrhotic liver tissues [28]. Downregulation of miR-24 in cirrhotic viral-associated HCC primary tissues is related to a worse prognosis. Low miR-101 expression is observed in HBV-associated HCC primary specimens, while the expression in serum is significantly elevated compared to healthy individuals [29]. MiR-145 and miR-199b are under-expressed and miR-224 is over-expressed in HBV-associated HCC patients [30]. A study by Pineau *et al.*, has shown that miR-22, miR-224, miR-34a, miR-425, miR-529, miR-93, and miR-96 are upregulated and let-7 is significantly downregulated during liver cancer progression [31]. Expression of particular microRNAs tends to change gradually during initiation and progression of liver cancer. Several tumor suppressor genes have been validated as target genes of hepatocellular carcinoma oncomirs, for example *TGFβ* receptors for miR-17-92 and *PTEN* for miR-21, miR-221, and miR-222. In contrast, oncogenes including *MYC* and *RAS* are direct targets of the miR let-7 family. In addition, *TCL-1* and *AKT* are validated as direct targets of miR-29, as well as *HNF1A* and *SRF* as target genes of miR-122 in HCC [25,26,27]. Several signaling pathways including Wnt/β-catenin, Ras, TGF-β, and JAK/STAT are among the prominent targets of microRNA dysregulation in HCC [24].

## 4. MicroRNAs as Diagnostic Markers

In the study of liver cancer, one of the ultimate goals is to develop early diagnostic markers, since HCCs are usually diagnosed at a late stage with severe liver dysfunction and limited therapeutic options. Current diagnostic markers available in the clinics still rely on AFP, routine USG and liver function tests [32]. MicroRNAs have emerged as potential diagnostic markers in HCC. A substantial number of studies have revealed microRNA dysregulation during initiation and progression of HCC. Those studies commonly compared expression of microRNA with healthy liver tissues or adjacent peritumoral tissues. Differential microRNA expression patterns can distinguish malignant from benign and pre-cancerous lesions. In addition, unique patterns of microRNA expression can discriminate malignant tumors into different molecular subtypes. One advantage of microRNAs as biomarkers is the stability in snap-frozen samples, archival formalin-fixed paraffin-embedded (FPPE) tissues, and body fluids including plasma/serum, urine, and saliva. The capability to measure microRNA expression in body fluids provides a remarkable opportunity for non-invasive early cancer diagnosis. One of the most important advantages of microRNA as HCC biomarkers is the fact that the dysregulation has been found in various specimens including primary tissues, blood, plasma, and urine. As invasive biopsies or surgery is required for collecting tissue samples, their use is not an ideal approach. Circulating microRNAs provide an alternative approach for tumor biomarkers. However, how patterns of circulating microRNAs represent the actual context of the tumor biology is still debated. The delivery into the extracellular space, the paths into circulation, and the processes to avoid ribonuclease degradation, and the contribution of normal and tumor cells toward microRNA release into circulation all affect the microRNA patterns found in the circulation and various body fluids. The release of free microRNA into circulation takes place both through passive and active processes. The passive microRNA release arises from defective cells of inflamed tissues, apoptosis or necrosis, while active microRNA release is commonly mediated through microvesicle. Lipoprotein complex, apoptotic bodies, and microvesicles mediate microRNA transport, which protects them from degradation.

### 4.1. Primary Tissue Specimens

Profiling studies using deep sequencing both in primary HCC specimens have established the basal microRNA expression patterns in hepatocytes and healthy liver, as well as in HCC [33,34]. The most abundantly expressed microRNA in liver is miR-122 (up to 50% of the total amount of microRNAs) and it is commonly down-regulated in HCC. MiR-199a/b is frequently downregulated in primary HCC samples and significantly associated with poor survival [34].

### 4.2. Serum

Contrary to findings in primary tumor samples, serum levels of miR-122 are unexpectedly higher in HCC patients compared to healthy individuals and the levels are significantly diminished after therapy [35]. The possible reason for the opposing levels between primary tumor and circulating samples is microRNA release from tumor cells into the circulation. The initial report from Li *et al.* [36] involving more than 500 serum samples from HCC patients showed that 13 microRNAs were differentially expressed in hepatitis B and hepatitis C virus-associated HCCs compared to healthy individuals. In total, 6 microRNAs were upregulated in the sera of HBV associated HCC samples. The combination of 3 microRNAs (miR-25, miR-375, and let-7f) was able to discriminate HCCs from controls. A single microRNA, miR-375, has receiver operating characteristic (ROC) of 0.96, with a sensitivity of 100% and a specificity of 96% in predicting liver cancer. Serum levels of miR-16, miR-195, and miR-199a, both alone and in combination, are able to discriminate HCC from chronic infection [37]. Compared to classic HCC markers such as AFP, DCP, and AFP-L3, miR-16 alone is the most sensitive marker to detect HCC. In HCC patients with lesions less than 3 cm, miR-16 performs better to detect the disease compared to the three classic markers [37]. Over-expression of miR-15b, miR-21, miR-130b, and miR-183 is documented in 96 tumors and the expression is significantly lower after surgery. These results indicate that circulating microRNAs most probably derive from the tumor cells [38]. Expression levels of miR-15b and -130 are able to detect HCC with sensitivity and specificity above 90%. A recent study by Lin *et al.*, has shown that a microRNA classifier consisting of 7 microRNAs (miR-29a, miR-29c, miR-133a, miR-143, miR-145, miR-192, and miR-505) can detect HCC at the time of diagnosis with better sensitivity than AFP (cut off 20 ng/mL) and similar specificity to AFP. In addition, the microRNA classifier is able to detect small, early stage, and AFP negative HCC. It can therefore serve as a preclinical parameter to detect HCC patients with the chance of curative resection and better survival [39].

### 4.3. Plasma

A study by Zhou *et al.* [40] using plasma samples from 934 HBV-associated HCC patients has revealed a microRNA panel with significant accuracy in detecting HCC. This panel was able to distinguish HCC from healthy, chronic HBV, and cirrhosis patients. The plasma levels of the miR-106 family have been shown to have ability to screen differentiated HCC from healthy individuals and that from patients with chronic liver disease [41]. Expression patterns of 4 microRNAs (miR-20a-5p, miR-320a, miR-324-3p and miR-375) have relative high sensitivity and specificity to differentiate HCC from non-cancerous liver lesions [42]. These studies demonstrate that circulating microRNAs are very promising candidates for non-invasive diagnostic markers in HCC. However, not a single microRNA overlaps between these two studies. Technical issues and source of materials (plasma *vs.* serum) might cause these differences.

In total, these data represent the feasibility of using circulating microRNA as a diagnostic marker in HCC. However, to translate these findings into clinical practice, more efforts are required for confirmation, including the best sample to be used (plasma, serum, or another body fluid), and comprehensive studies involving prospective multicenter trials to evaluate the power of circulating microRNA as a new diagnostic biomarker.

## 5. MicroRNA Profiling for Prognosis in HCC

In addition to their potential as a marker for diagnosis and monitoring therapy, microRNAs can also be used as prognostic markers in HCC. Differential expression of microRNAs is often associated with TNM stage (size, nodal and distant metastasis), tumor invasion, recurrence, and overall survival. Su *et al.* demonstrated upregulation of miR-25 in primary HCC tissues and a significant association with the TNM stage [43]. Upregulation of miR-183 [44] and miR-17-5p [45] in primary HCC specimens after surgical resection has also been associated with larger tumor size, positive nodal status and higher propensity for distant metastasis. In non-metastatic HCC, primary tissue expression of miR-17-5p is significantly lower and is associated with elevated *E2F1* expression [45,46]. In addition, high miR-221 expression in primary tissues is frequently shown in HCC with distant metastasis [47]. Decreased miR-100 and miR-22 expression levels in primary HCC tissues correlate with progressive pathological features [48,49]. Downregulation of miR-338 in cancerous tissues is significantly associated with higher TNM stage, vascular invasion and intrahepatic metastasis [50]. Portal vein invasion, distant metastasis, and higher TNM stage in HCC have also been correlated with downregulation of miR-34a [51], miR-148a [52], miR-101, miR-148b and miR-214 in primary tumor tissues [53,54,55]. Expression patterns of 31 microRNAs in primary tissues can differentiate the clinical HCC stages [56]. In addition, the expression patterns of 20 microRNAs in tissue specimens are associated with distant metastasis in HCC [57]. Using sera from 46 HCC patients and controls, Li *et al.* [58] have determined that miR-221 is upregulated in patient’s sera and is significantly associated with tumor size, cirrhosis, and tumor stage. Upregulation miR-222 [58] in patient’s sera are also correlated with advanced tumor stage. In addition, progression of HCC could be monitored by plasma expression of miR-21 expression in HCC. Decreased expression of plasma miR-21 has been shown in HCC after receiving standard therapy [59].

Deregulation of microRNAs is also associated with HCC survival. Upregulation of miR-25 [43], miR-372 [60], miR-155 [61], and miR-182 [62] in primary tissue specimens is significantly correlated with shorter overall survival. In addition, downregulation of miR-29a-5p [63], miR-100 [47], miR-29 [64], miR-101 [53], miR-148a [54] in primary HCC tissues is associated with reduced freedom from disease and overall survival. Expression patterns of miR-19a, miR-886-5p, miR-126, miR-223, miR-24, and miR-147 in primary tissue HCC samples also correlate with overall survival following liver transplantation [65]. Overall survival of HCC patients with elevated plasma miR-221 is worse compared to those without any expression change or dowregulation [58].

Expression of microRNAs has also been inferred to predict disease-recurrence after completion of therapy. In primary HCC tissues, upregulation of miR-155 [61] and miR-221 [47] correlates with frequent recurrence. Decreased expression of miR-29a-5p [63] and miR-214 [55] in tumor tissues is associated with early HCC recurrence.

## 6. MicroRNAs as Therapeutic Targets and for Monitoring Therapeutic Response

Recent studies have revealed the potential application of miRNAs as therapeutic targets in HCC. The unique biological mechanisms by which miRNAs fine-tune gene expression during liver cancer development provide novel targets for therapeutic intervention as well as posing some challenges for the development of new drugs. For cancer therapy, miRNA antagonists are used to block oncogenic microRNAs (oncomir). Several antagonists, including locked nucleid acid (LNA) or antagomirs with different modifications, have been studied both *in vivo* and *in vitro*. MicroRNA antagonists inhibit oncomirs through complementary base-pairing with some chemical modifications to improve binding affinity, hinder nuclease degradation, and foster cellular uptake [66].

Suppression of oncogenic miR-221 resulted in better overall survival and significantly decreased tumor number and size in an animal model [67]. In the case of tumor suppressor microRNA downregulation in HCC patients, reintroduction of microRNA mimics has also been studied. The challenge for microRNA mimics is the delivery to the tumor site since systemic introduction might produce off-target effects. Delivery using viral vector systems has been studied by Kota *et al.* They delivered mir-26 systematically in a mouse model that resulted in inhibition of cell proliferation and induction of tumor-specific apoptosis [68]. A phase III clinical trial with anti-miR-122 (miravirsen) for chronic HCV infection has been initiated [69]. In addition, a phase I clinical trial using liposome-based miR-34 mimics has also been conducted [70]. Further larger clinical trials are required to assess the application of microRNA based therapy in HCC.

In HCC, interferon is one of the most frequently used drugs to improve survival. A recent study in IFN-resistant HCC cells showed that miR-146a influenced response to interferon therapy in HCC. Upregulation of miR-146a led to *SMAD4* downregulation and conferred resistance to interferon [71]. On the other hand, low tissue expression of miR-26 was associated with improved response to interferon [72] with significant better overall survival [73]. Transfection of anti-miR-21 in HCC cell lines leads to better response to combination chemotherapy using interferon-α and 5-FU [74].

Targeted therapy that is commonly used in HCC management, *i.e.*, administration of sorafenib, has also been reported to regulate microRNA expression. MicroRNA expression analysis can be performed via fine-needle aspiration before administering sorafenib, and specific patterns might predict response to therapy. Fourteen microRNAs including miR-1274 are upregulated upon sorafenib treatment in HCC cell lines causing *ADAM9* downregulation. ADAM9 is a protease involved in sorafenib-mediated response in HCC [75] MiR-122 is commonly downregulated in HCC. Restoration of miR-122 expression in HCC cells leads to increased sensitivity upon sorafenib treatment [76]. Upregulation of miR-338 in HCC cells significantly correlates with increased response to sorafenib [77]. MiR-34a that is frequently downregulated in HCC targets Bcl2 and is able to sensitize HCC cells to sorafenib treatment. Low expression of miR-34a might predict sorafenib resistance [78].

In response to chemotherapy, forced expression of miR-122 in HCC cells leads to increased sensitivity to certain drugs including doxorubicin [79]. Re-expression of miR-122 in HCC cells also induces sensitivity to adriamycin and vincristine through reduced expression of multidrug resistance (MDR) proteins such as ABC, anti-apoptotic Bcl-w and cyclin B1 [80]. Expression levels of miR-199a-3p influence the sensitivity of HCC cells to doxorubicin [81,82]. Zhao *et al.*, have documented that miR-26b hinders NF-κB signaling and the overexpression is correlated with significantly increased sensitivity of HCC cells to doxorubicin. In HCC cells, overexpression of miR-101 correlated with autophagy inhibition and cisplatin-induced apoptosis [83]. Inhibition of miR-199a-3p expression through DNA methylation confers resistance to 5-fluorouracil. To predict therapeutic response, promoter DNA methylation and expression of miR-193a-3p represent useful markers for resistance to 5-FU treatment through repression of SRSF2 expression [84]. Overexpression of miR-27, which targets MDR1/P-glycoprotein and β-catenin, is a predictor for therapeutic response to 5-fluorouracil [85]. In addition, high expression of miR-141 predicts resistance of HCC cells to 5-fluorouracil [86]. MiR-23a inhibits topoisomerase expression and therefore its upregulation might predict the response to etoposide in HCC [87]. MiR-26b targets NF-κB regulators *TAK1* and *TAB3* to mediate chemosensitivity [88]. Differential expression of protein expression of drug transporters has long been associated with chemotherapeutic resistance. A study in HCC cell lines showed that downregulation of miR-223 led to multidrug resistance since miR-223 targeted *ABCB1* expression [89]. Borel *et al.* [90] have shown that 13 microRNAs regulate expression of adenosine triphosphate-binding cassette (ABC) transporters and mediate chemotherapeutic resistance in HCC.

## 7. Future Directions

Although application of microRNA as biomarkers for diagnosis, prognosis, and monitoring therapy in liver cancer is very promising, several problems still need to be addressed. For application in routine clinical practice, techniques used for measurement of microRNA expression have to be standardized. Array or deep sequencing technology is relatively expensive and inter-laboratory variability is still a major challenge. Quantification of selected microRNAs using quantitative reverse-transcriptase PCR or multiplex bead-based quantification will be economically applicable and much easier to standardize. In terms of determining which microRNA panels will be used as a biomarker in HCC, no universal consensus has been reached so far. Most studies addressing biomarkers in HCC use samples from Asian populations with primarily virus-associated HCC cases [1]. Although European and American HCC cases are associated with viruses, recent trends show that NAFLD-related HCCs are increasing [91]. These differences might result in different microRNA panels useful for prognosis and therapeutic monitoring. In addition, due to the complexity of microRNA roles during hepatocarcinogenesis, for some microRNAs a huge discrepancy exists between different studies. Cancer molecular heterogeneity, different response of the tumor microenvironment, and technical issues might underlie this inconsistency.

Almost all HCC cases are found in patients with moderate or severe liver dysfunction. Patterns of microRNA expression both in primary HCC specimens and circulating samples can be influenced by liver dysfunction independent of the biology of liver cancer [92]. The inverse correlation between tissue and circulating microRNA expression could be affected by the pathology of liver dysfunction. In addition, we also still have to determine which sample provides the best reliable result for HCC biomarkers [92]. Specimens from primary tumor samples represent the actual biology of tumor development and progression. However, acquiring 100% pure tumor tissue for microRNA analysis is nearly impossible due to cellular contamination from the tissue microenvironment and circulating blood cells. For a non-invasive approach, either plasma or serum is a very promising source. Although they show great potential as biomarkers in HCC, a major constraint for the clinical application of microRNA measurements is the lack of standardization. The best and most cost-effective methods for microRNA quantification and normalization have yet to be determined to reduce interlaboratory variability. For normalization, utilization of more than two stable reference transcripts according to sample types is strongly recommended. In addition, the influence of preanalytical conditions (time and circumstances of samples collection, transport conditions, *etc.*) have to be evaluated more thoroughly and standardized in future trials.

MicroRNA expression profiling has great potential for the development of new clinical markers for HCC diagnosis, prognosis, and therapy monitoring, as summarized in Table 1. However, multi-center studies incorporating different panels of microRNAs and using various clinical stages of HCC patients are required to validate them as clinical biomarkers. Studies involving large clinical cohorts within a population-based setting are required.

**Table 1:** MicroRNAs as diagnostic, prognostic, and therapy monitoring markers in HCC.

Diagnostic Biomarkers				
MicroRNA	Regulation	Source	Information	Ref
miR-106	Up	Plasma	Differentiate HCC from healthy control and chronic liver disease	[41]
miR-122	Up	Serum	Differentiate HCC from healthy control	[35]
miR-15b, miR-130b	Up	Serum	Differentiate HCC from healthy control	[38]
miR-16, miR-199a	Down	Serum	Differentiated HCC from chronic hepatitis and healthy control	[37]
miR-183	Up	Tissue	Differentiate benign and malignant liver tumor	[39]
miR-15b, miR-130b	Up	Serum	Differentiate HCC and healthy patients and reduce after surgery	[38]
miR-18a	Up	Serum	Differentiate HCC and healthy patients	[93]
miR-122, miR192, miR-21, miR-223, miR-26a, miR-27a, miR-801	Signature	Plasma	Differentiated HCC from cirrhosis, chronic liver patients, and healthy controls	[40]
miR-21	Up	Serum, plasma	Differentiate HCC from cirrhosis and healthy controls	[59,94]
miR-375	Up	Serum	Differentiated HBV- and HCV-related HCC from healthy controls	[36]
miR-483	Up	Plasma	Differentiated HCC patients from healthy controls	[95]
miR-618/650	Up	Urine	Differentiate HCC and control	[96]
miR-885	Up	Serum	Differentiate HCC, cirrhosis, and chronic liver patients from healthy controls	[97]
miR-92a	Down	Plasma	Differentiated HCC from healthy control	[98]
miR-25, miR-375, let-7f	Up	Serum	Differentiate HCC from healthy control	[36]
miR-20a-5p, miR-320a, miR-324-3p and miR-375	Up	Plasma	Differentiate HCC from non-cancerous lesions	[42]
miR-29a, miR-29c, miR-133a, miR-143, miR-145, miR-192, and miR-505	Signature	Serum	Detect early stage HCC and AFP-negative HCC	[99]
**MicroRNAs as Prognostic Markers**				
miR-10b	Up	Tissue	Poor prognosis	[100]
miR-122	Down	Tissue	Poor prognosis	[101]
miR-124	Down	Tissue	Poor prognosis and aggressive type	[102]
miR-135a	Up	Tissue	Shorter overall survival and disease-free survival	[103]
miR-139	Down	Tissue	Metastasis and poor prognosis	[104]
miR-155	Up	Tissue	Poor prognosis, recurrence, micro-vascular invasion	[61]
miR-182	Up	Tissue	Intrahepatic metastasis and poor prognosis	[62]
miR-199b-5p	Down	Tissue	Shorter overall survival	[105]
miR-203	Up	Tissue	Better prognosis, longer survival	[106]
mi-21, miR-221	Up	Tissue	Tumor stage and poor prognosis	[107]
miR-22	Down	Tissue	Poor survival	[108]
miR-221	Up	Serum	Poor survival	[58]
miR-29	Down	Tissue	Shorter disease-free survival	[64]
miR-29a-5p	Up	Tissue	Recurrence in early stage HCC	[63]
miR-99a	Down	Tissue	Shorter survival	[109]
let-7g	Down	Tissue	Poor survival	[110]
DLK1-DIO3 miRNA cluster	Up	Tissue	Poor prognosis	[111]
C19MC microRNA cluster	Up	Tissue	Poor clinico-pathological features, recurrence, and shorter overall survival	[112]
miR-155, miR-15a, miR-432, miR-486-3p, miR-15b, miR-30b	Up	Tissue	Recurrence-free survival	[113]
miR-19a, miR-886, miR-126, miR-223, miR-24, and miR-147	Signature	Tissue	Overall survival and recurrent free survival	[65]
67 miRs signature	Signature	Tissue	Differentiate recurrence after liver transplantation	[114]
miR signatures in tumor and non-tumor tissues	Signature	Tissue	Differentiate early and late recurrence	[115]
miR-326, miR-3677, miR-511-1, miR-511-2, miR-9-1, and miR-9-2	Signature	Tissue	Negatively associated with overall survival	[116]
**Predictive Therapeutic Response Markers**				
miR-122	Down	Cells, tissue	Decreased sensitivity to Doxorubicin	[81]
miR-122	Down	Cells, tissue	Decreased sensitivity to Adriamycin, Vincristin	[80]
miR-122	Down	Cells, tissue	Suppressed sensitivity to sorafenib	[76]
miR-146a	Up	Cells	Suppresses sensitivity to interferon-α	[71]
miR-193a-3p	Down	Cells, tissue	Resistance to 5-FU	[84]
miR-193b	Up	Cells, Tissue	Sensitivity to cisplatin	[117]
miR-199a-3p	Down	Cells, tissue	Increased sensitivity to Doxorubicin	[82]
miR-1247a	Down	Cells	Resistance to sorafenib	[118]
miR-21	Up	Cells, tissue	Resistance to interferon-α/5FU in HCC cells	[74]
miR-34a	Down	Cells, tissue	Resistance to sorafenib	[94]
13 microRNA signature	Signature	Cells, tissue	Multidrug resistance	[90]
